# Supplementary material for: Hierarchical organization of rhesus macaque behavior
Source: Oxf Open Neurosci. 2023 Jun 20;2:kvad006. doi: 10.1093/oons/kvad006 (PMC10421634; doi:10.1093/oons/kvad006)
Supplement: Web_Material_kvad006 [file Web_Material_kvad006.docx]

**REVIEWER COMMENTS**

**OXFNSC-2023-003.R1 - "Hierarchical organization of rhesus macaque behavior"**

**Original submission - OXFNSC-2023-003**

**Reviewer 1**

Comments to Author

In this manuscript, the authors use a complex multi-camera tracking system and sophisticated computational analyses to study the movement behavior of two rhesus macaques under three different behavioral contexts. The report that the continuum of subjects’ behavior can be broken down into a a relatively small number of joint-angle configurations (postures) that frequently recur across different positions within the environment. Postures are in turn organized hierarchically, with transitions between behaviors within the same module more frequently observed than transitions between modules.

Overall, there is a lot of like here. The data the authors analyze here depends on an expensive, specialized experimental setup; three-dimensional pose data from unrestrained macaques makes for a unique and valuable data set. The analyses are sophisticated, but generally well described and fairly easy to follow. Importantly, the analyses described here should in principle generalize to any data set consisting of multipoint tracking, which can be obtained in some systems using simpler hardware setups than the authors were forced to employ here to track macaques. The analytical pipeline here complements similar work in rodents, but identifies postures and modules in an entirely unsupervised way, which contrasts with approaches that require user input to set the timescale over which behavioral sequences are identified. So the approach used here achieves similar ends but with some different steps along the way, which makes it a potentially valuable contribution to the emerging area of computational neuroethology.

Below, I have a few questions to resolve some areas of confusion, and a few relatively minor suggestions for improving the manuscript further.

Main questions:

I follow the analysis pipeline generally, but there are two aspects that aren’t entirely clear to me. Given that these methods are likely to be broadly useful across a range of species some further details would be helpful. First, what exactly is the alignment across data sets achieving. The authors state this was done in analogy to correcting for batch effects in genomics data, but I’m not familiar with the problem that arises in that instance or how it is solved here. Further clarity would be appreciated.

Similarly, I don’t understand the use of UMAP here. Could the authors provide additional explanation of why this step is necessary? It seems like the data has already been through dimensionality reduction via PCA at this point. Would it not be possible to cluster poses in PC space? I assume the answer is no, but I’d like to better understand why not, and how UMAP gets around the problem.

Minor issues:

For analysis of the timescale of individual subjects’ behavior, it seems like the authors are using a two way anova that doesn’t account for the fact that data from the same subject are not independent. There’s a pretty complicated nesting situation here, with data coming from individual animals in individual sessions, but with some shared types of sessions. The statistical approach should properly account for these dependencies.

There are several points in the manuscript where the authors’ tone and phrasing in describing the utility of this work could give readers incorrect impressions. For instance, they correctly note that similar approaches in rodents have identified repeated behavioral units that are organized into motifs. They argue that it is unclear whether similar principles hold in “larger animals with more complicated behavioral repertoires”. I’m sure the authors didn’t mean to imply that the size of an organism in some way determines the complexity of its motor behavior, but a reader could get this impression.

More importantly, I’m not sure there is a universally agreed up definition of complexity with respect to either individual behaviors or an organism’s behavioral repertoire. I would advise caution in comparing behaviors or organisms in terms of complexity without some explicit definition, as it can easily evokes unscientific notions of a scala naturae, with primates like humans at the top as the teleological goal of evolution.

Finally, I am unconvinced that the higher dimensionality of macaque bodies seriously calls into question whether their behavior would obey similar organizing principles identified in other species. Setting aside the issue that the dimensionality of a measured behavior depends on both the organism, the measurement technique (had the authors been able to track the individual digits of their subjects they would have achieved a more high-dimensional readout of their subjects’ behavior) and the environmental affordances of a particular behavioral context, it seems that a having a higher-dimensional body that nevertheless must be controlled by a finite number of neurons would make it even more advantageous for behavior to live in a lower dimensional, modular space!

In a similar vein, there are places where the authors overstate their conclusions. Although this is an impressive data set, it is nevertheless two individuals, both male, in three behavioral contexts. So a figure title like “Behavioral modularity is universal and unique” seems like it requires quite a massive dose of qualification to be accurate. It would be nice to have more discussion of the limitations of the present data set throughout the manuscript. For instance, the authors are making claims about detecting individual differences in movement grammar here. But their data set is only two individuals! How far can we generalize this result? If the point of the result is to show that the method is capable of detecting common and individually idiosyncratic features of behavior, fair enough. But it should be acknowledged that it is currently hard to interpret what the idiosyncrasies mean, or how they would be distributed across a larger population of subjects.

**Reviewer 2**

Comments to Author

Authors present an analysis of 3D poses in 2 macaques, acquired using 15 landmarks as macaques move freely in a large open space. Authors used an unsupervised method to identify 49 unique postures and clustered them into modules that in turn were organized hierarchically, ultimately into higher-level action sequences. Behaviors were more stable on task, than off. Behavior modules and timescales were organized and stable within individuals, more than across task.

Perhaps one of the more intriguing findings of the present report is that constraint of behavior by environmental demands (provided by the task) are not due to a narrowing of behavioral repertoire during the task but rather that the actions themselves become less variable. Additionally, that pose data can be used to infer higher-level categories from the elemental unit of postures (and their transitions) is valuable information. The paper was clearly written and the data visualization was quite good. I have only a couple of major comments, and very minor suggestions for enhancing clarity.

Of the 23 features, 19 were joint landmarks and 4 were related to speed and a PCA was applied to these data to determine the features “used in subsequent embedding and clustering” (lines 504-505). However, the authors then go on to describe the UMAP where parameters were set, in my opinion, rather qualitatively because they were “found to be a good balance between separating dissimilar poses, while combining similar ones.” I worry that this description will not yield reproducible findings by other groups trying to use this method. In fact, it is not clear what the UMAP achieves beyond the PCA, please clarify.

I was surprised to find that within-subject and between-subject module stability was analyzed via unpaired t-test when the data are collapsed and thus not independent. Similarly, a 2-way ANOVA on timescale across behavior does not seem appropriate. A multi-level generalized linear model with all between and within-subject factors included in the model would be more appropriate.

In general, I was disappointed with the lack of rodent behavioral tracking citations. I understand this is a macaque paper, but much has been learned from other species for behavioral elements/motifs and transitions to coordinated movement, or “motor sequences de novo” (Olveczky, Curr Op in Neurobio). Additionally, Luxem et al. could be cited https://elifesciences.org/articles/79305

Minor

Line 24 “However, precise measure of behavior [in macaques] has generally been limited…”

Line 41 “macaque behaviors might not obey the same principles” as what?

Line 240 p=0.039

**Decision letter - OXFNSC-2023-003**

| 19-May-2023  Dear Dr. Hayden,  Manuscript ID OXFNSC-2023-003 entitled "Hierarchical organization of rhesus macaque behavior" which you submitted to the Oxford Open Neuroscience, has been reviewed. The comments of the reviewers are included at the bottom of this letter.  The reviewers suggest some revisions to your manuscript. Therefore, I invite you to respond to these comments and revise and resubmit your manuscript.  Please note that this journal operates with transparent peer review. This means that if your submission is accepted for publication, the full peer review history of your article will publish online alongside your article. This includes reviewer comments, editor decision letters, and your author responses.  To revise your manuscript, log into https://mc.manuscriptcentral.com/oxfnsc and enter your Author Centre, where you will find your manuscript title listed under "Manuscripts with Decisions."  Under "Actions," click on "Create a Revision."  Your manuscript number has been appended to denote a revision.  You may also click the below link to start the revision process (or continue the process if you have already started your revision) for your manuscript. If you use the below link you will not be required to login to ScholarOne Manuscripts.  *** PLEASE NOTE: This is a two-step process. After clicking on the link, you will be directed to a webpage to confirm. ***  https://mc.manuscriptcentral.com/oxfnsc?URL_MASK=eed939dd8f9b456392a963982cf0ce5f  You will be unable to make your revisions on the originally submitted version of the manuscript.  Instead, revise your manuscript using a word processing program and save it on your computer.  Please also highlight the changes to your manuscript within the document by using the track changes mode in MS Word or by using bold or colored text.  Once the revised manuscript is prepared, you can upload it and submit it through your Author Centre.  When submitting your revised manuscript, you will be able to respond to the comments made by the reviewers in the space provided.  You can use this space to document any changes you make to the original manuscript.  In order to expedite the processing of the revised manuscript, please be as specific as possible in your response to the reviewers.  IMPORTANT:  Your original files are available to you when you upload your revised manuscript.  Please delete any redundant files before completing the submission.  Because we are trying to facilitate timely publication of manuscripts submitted to the Oxford Open Neuroscience, your revised manuscript should be uploaded as soon as possible.  If it is not possible for you to submit your revision in a reasonable amount of time, we may have to consider your paper as a new submission.  Once again, thank you for submitting your manuscript to the Oxford Open Neuroscience and I look forward to receiving your revision.  Sincerely, Dr. Alicia Izquierdo Senior Editor, Oxford Open Neuroscience aizquie@psych.ucla.edu  **Reviewer: 1** Comments to the Author In this manuscript, the authors use a complex multi-camera tracking system and sophisticated computational analyses to study the movement behavior of two rhesus macaques under three different behavioral contexts. The report that the continuum of subjects’ behavior can be broken down into a a relatively small number of joint-angle configurations (postures) that frequently recur across different positions within the environment. Postures are in turn organized hierarchically, with transitions between behaviors within the same module more frequently observed than transitions between modules.  Overall, there is a lot of like here. The data the authors analyze here depends on an expensive, specialized experimental setup; three-dimensional pose data from unrestrained macaques makes for a unique and valuable data set. The analyses are sophisticated, but generally well described and fairly easy to follow. Importantly, the analyses described here should in principle generalize to any data set consisting of multipoint tracking, which can be obtained in some systems using simpler hardware setups than the authors were forced to employ here to track macaques. The analytical pipeline here complements similar work in rodents, but identifies postures and modules in an entirely unsupervised way, which contrasts with approaches that require user input to set the timescale over which behavioral sequences are identified. So the approach used here achieves similar ends but with some different steps along the way, which makes it a potentially valuable contribution to the emerging area of computational neuroethology.  Below, I have a few questions to resolve some areas of confusion, and a few relatively minor suggestions for improving the manuscript further.  Main questions:  I follow the analysis pipeline generally, but there are two aspects that aren’t entirely clear to me. Given that these methods are likely to be broadly useful across a range of species some further details would be helpful. First, what exactly is the alignment across data sets achieving. The authors state this was done in analogy to correcting for batch effects in genomics data, but I’m not familiar with the problem that arises in that instance or how it is solved here. Further clarity would be appreciated.  Similarly, I don’t understand the use of UMAP here. Could the authors provide additional explanation of why this step is necessary? It seems like the data has already been through dimensionality reduction via PCA at this point. Would it not be possible to cluster poses in PC space? I assume the answer is no, but I’d like to better understand why not, and how UMAP gets around the problem.  Minor issues:  For analysis of the timescale of individual subjects’ behavior, it seems like the authors are using a two way anova that doesn’t account for the fact that data from the same subject are not independent. There’s a pretty complicated nesting situation here, with data coming from individual animals in individual sessions, but with some shared types of sessions. The statistical approach should properly account for these dependencies.  There are several points in the manuscript where the authors’ tone and phrasing in describing the utility of this work could give readers incorrect impressions. For instance, they correctly note that similar approaches in rodents have identified repeated behavioral units that are organized into motifs. They argue that it is unclear whether similar principles hold in “larger animals with more complicated behavioral repertoires”. I’m sure the authors didn’t mean to imply that the size of an organism in some way determines the complexity of its motor behavior, but a reader could get this impression.  More importantly, I’m not sure there is a universally agreed up definition of complexity with respect to either individual behaviors or an organism’s behavioral repertoire. I would advise caution in comparing behaviors or organisms in terms of complexity without some explicit definition, as it can easily evokes unscientific notions of a scala naturae, with primates like humans at the top as the teleological goal of evolution.  Finally, I am unconvinced that the higher dimensionality of macaque bodies seriously calls into question whether their behavior would obey similar organizing principles identified in other species. Setting aside the issue that the dimensionality of a measured behavior depends on both the organism, the measurement technique (had the authors been able to track the individual digits of their subjects they would have achieved a more high-dimensional readout of their subjects’ behavior) and the environmental affordances of a particular behavioral context, it seems that a having a higher-dimensional body that nevertheless must be controlled by a finite number of neurons would make it even more advantageous for behavior to live in a lower dimensional, modular space!  In a similar vein, there are places where the authors overstate their conclusions. Although this is an impressive data set, it is nevertheless two individuals, both male, in three behavioral contexts. So a figure title like “Behavioral modularity is universal and unique” seems like it requires quite a massive dose of qualification to be accurate. It would be nice to have more discussion of the limitations of the present data set throughout the manuscript. For instance, the authors are making claims about detecting individual differences in movement grammar here. But their data set is only two individuals! How far can we generalize this result? If the point of the result is to show that the method is capable of detecting common and individually idiosyncratic features of behavior, fair enough. But it should be acknowledged that it is currently hard to interpret what the idiosyncrasies mean, or how they would be distributed across a larger population of subjects.  **Reviewer: 2** Comments to the Author Authors present an analysis of 3D poses in 2 macaques, acquired using 15 landmarks as macaques move freely in a large open space. Authors used an unsupervised method to identify 49 unique postures and clustered them into modules that in turn were organized hierarchically, ultimately into higher-level action sequences. Behaviors were more stable on task, than off. Behavior modules and timescales were organized and stable within individuals, more than across task.  Perhaps one of the more intriguing findings of the present report is that constraint of behavior by environmental demands (provided by the task) are not due to a narrowing of behavioral repertoire during the task but rather that the actions themselves become less variable. Additionally, that pose data can be used to infer higher-level categories from the elemental unit of postures (and their transitions) is valuable information. The paper was clearly written and the data visualization was quite good. I have only a couple of major comments, and very minor suggestions for enhancing clarity.  Of the 23 features, 19 were joint landmarks and 4 were related to speed and a PCA was applied to these data to determine the features “used in subsequent embedding and clustering” (lines 504-505). However, the authors then go on to describe the UMAP where parameters were set, in my opinion, rather qualitatively because they were “found to be a good balance between separating dissimilar poses, while combining similar ones.” I worry that this description will not yield reproducible findings by other groups trying to use this method. In fact, it is not clear what the UMAP achieves beyond the PCA, please clarify.  I was surprised to find that within-subject and between-subject module stability was analyzed via unpaired t-test when the data are collapsed and thus not independent. Similarly, a 2-way ANOVA on timescale across behavior does not seem appropriate. A multi-level generalized linear model with all between and within-subject factors included in the model would be more appropriate.  In general, I was disappointed with the lack of rodent behavioral tracking citations. I understand this is a macaque paper, but much has been learned from other species for behavioral elements/motifs and transitions to coordinated movement, or “motor sequences de novo” (Olveczky, Curr Op in Neurobio). Additionally, Luxem et al. could be cited https://elifesciences.org/articles/79305  Minor Line 24 “However, precise measure of behavior [in macaques] has generally been limited…” Line 41 “macaque behaviors might not obey the same principles” as what? Line 240 p=0.039  Associate Editor: 1 Comments to the Author: There appears to be consensus from both Reviewers on the lack of clarity of (rationale for) the UMAP and perhaps some concerns about the choice of statistical tests for nested data. Additionally, Reviewer 1 asking for more clarity on the rationale for alignment across datasets is justified. Reviewer 1 also raises a fair point about tempering bold claims with some stated limitations of the present approach. And finally Reviewer 2's suggestion to include rodent behavioral tracking citations seems reasonable. Overall, this is a valuable contribution to the study of naturalistic behavior in primates.  Date Sent: 19-May-2023 |
| --- |

**Author response**

**Reviewer: 1**
I follow the analysis pipeline generally, but there are two aspects that aren’t entirely clear to me. Given that these methods are likely to be broadly useful across a range of species some further details would be helpful. First, what exactly is the alignment across data sets achieving. The authors state this was done in analogy to correcting for batch effects in genomics data, but I’m not familiar with the problem that arises in that instance or how it is solved here. Further clarity would be appreciated.

- The reviewer poses a very crucial question that we had unfortunately not properly explained in the manuscript. Essentially, embedding approaches such as t-SNE, UMAP, and diffusion embedding are arbitrary in their embedding space which means that even slight variability between datasets can lead to large differences in the embedding space which will make interpretability difficult. These problems can be greatly reduced by proper alignment of the data before embedding.

- This problem is acute in genomics data analysis, and as a consequence, these researchers have developed techniques that can help us as well. Specifically, they have devised specific baseline and noise offsets that we used. We have added the following explanation to the manuscript:

"Alignment is required to ensure that idiosyncratic variability in behavior does not bias the embedding process. This alignment serves the function of correcting for small day to day measurement effects such as the distribution of poses and transitions present in daily measurements. These effects, while small, can wind up having very large spurious effects on the resulting clustering."

Similarly, I don’t understand the use of UMAP here. Could the authors provide additional explanation of why this step is necessary? It seems like the data has already been through dimensionality reduction via PCA at this point. Would it not be possible to cluster poses in PC space? I assume the answer is no, but I’d like to better understand why not, and how UMAP gets around the problem.

- Again this is a great comment and we apologize for the lack of clarity on our part.

- The UMAP procedure implements the data structures for clustering, that is, unsupervised identification of states. PCA on its own does not do this because it can only detect orthogonal components, and therefore misses a large amount of variance in the data. In other words, PCA is unsuited for this purpose.

- Instead, the purpose of the initial PCA is to improve computational efficiency. Essentially, most embedding solutions require pairwise comparison of data points making the computational complexity incredibly large. This problem can be reduced greatly with little loss of information by using PCA to impose sparsity on the data. A second benefit is that, since behavior is inherently lower dimensional than the data acquired, PCA additionally helps with noise reduction in the estimates.

- We have added the following explanation to the manuscript:

"Initial PCA dimensionality reduction was performed to reduce noise as well as to sparsen the data, which greatly increases computational efficiency."

- We have also added the following sentence:

"The PCA procedure serves to sparsen the data and reduce noise; the UMAP serves to implement the high dimensional clustering."

Minor issues:

For analysis of the timescale of individual subjects’ behavior, it seems like the authors are using a two way anova that doesn’t account for the fact that data from the same subject are not independent. There’s a pretty complicated nesting situation here, with data coming from individual animals in individual sessions, but with some shared types of sessions. The statistical approach should properly account for these dependencies.

- We thank the reviewer for this comment. As in primate neurophysiology, we treat sessions as independent measures. We do this partly out of necessity - we simply do not have access to the dozens of monkey subjects that would be needed if the subject were the unit of analysis. We also do this because the assumption is at least somewhat justified - the behavior does vary across days. However, it is worth noting that this assumption does reduce the conclusions that can be drawn from the data - they are not the same conclusions that could be drawn with a much larger number of subjects. Right now, we are much more interested in especially testing consistencies which our approach captures. Finally, it is also worth noting that this limitation does not risk false positives in our claims.

- We have added the following sentences to specifically point out this limitation of our study:

"Note that this analysis assumes that each session is independent of each other. We make this assumption out of necessity (because our number of subjects is low), and it is a limitation of the present work. Due to the limited number of sessions and animals, our study can not capture individual differences or model our findings in a mixed effects approach. We hope that over time our sample size will increase to specifically address these shortcomings."

- And also:

"Having said that, with only two subjects in our sample, the generality of this cross-individual claim is severely limited."

There are several points in the manuscript where the authors’ tone and phrasing in describing the utility of this work could give readers incorrect impressions. For instance, they correctly note that similar approaches in rodents have identified repeated behavioral units that are organized into motifs. They argue that it is unclear whether similar principles hold in “larger animals with more complicated behavioral repertoires”. I’m sure the authors didn’t mean to imply that the size of an organism in some way determines the complexity of its motor behavior, but a reader could get this impression.

- We have now gone through the manuscript to fix the tone and phrasing.

- For the particular concern raised (about size and complexity of behavior), we now no longer include this specific claim.

More importantly, I’m not sure there is a universally agreed up definition of complexity with respect to either individual behaviors or an organism’s behavioral repertoire. I would advise caution in comparing behaviors or organisms in terms of complexity without some explicit definition, as it can easily evokes unscientific notions of a scala naturae, with primates like humans at the top as the teleological goal of evolution.

- We apologize for these unintended implications. We have now removed these ideas from the manuscript, which are not essential for the core ideas of the paper.

Finally, I am unconvinced that the higher dimensionality of macaque bodies seriously calls into question whether their behavior would obey similar organizing principles identified in other species. Setting aside the issue that the dimensionality of a measured behavior depends on both the organism, the measurement technique (had the authors been able to track the individual digits of their subjects they would have achieved a more high-dimensional readout of their subjects’ behavior) and the environmental affordances of a particular behavioral context, it seems that a having a higher-dimensional body that nevertheless must be controlled by a finite number of neurons would make it even more advantageous for behavior to live in a lower dimensional, modular space!

- As with the previous comment, these ideas are not central to our case. We have removed these ideas from this manuscript.

In a similar vein, there are places where the authors overstate their conclusions. Although this is an impressive data set, it is nevertheless two individuals, both male, in three behavioral contexts. So a figure title like “Behavioral modularity is universal and unique” seems like it requires quite a massive dose of qualification to be accurate. It would be nice to have more discussion of the limitations of the present data set throughout the manuscript. For instance, the authors are making claims about detecting individual differences in movement grammar here. But their data set is only two individuals! How far can we generalize this result? If the point of the result is to show that the method is capable of detecting common and individually idiosyncratic features of behavior, fair enough. But it should be acknowledged that it is currently hard to interpret what the idiosyncrasies mean, or how they would be distributed across a larger population of subjects.

- We agree with these suggestions. We have now revised the manuscript to reduce the size of conclusions. In addition, we now include a new paragraph listing the limitations of our work.

"One major limitation of the present work is that we only recorded data in two subjects. As such we cannot make major claims about individual differences. Indeed, in our statistical analyses, we use the behavioral session, not the subject, as the unit of analysis. This approach risks reducing statistical power because it treats variance between subjects as no different from variance within. Another result of this limitation is that we have likely failed to explore the full range of possible behaviors and the diversity of behavioral organizations. As such, our general claims must be taken as preliminary. We believe that future work will mitigate some of these problems by including larger numbers of research subjects."

- And

"Having said that, with only two subjects in our sample, the generality of this cross-individual claim is severely limited."

- Finally, we also have rephrased the figure title.

**Reviewer: 2**
Of the 23 features, 19 were joint landmarks and 4 were related to speed and a PCA was applied to these data to determine the features “used in subsequent embedding and clustering” (lines 504-505). However, the authors then go on to describe the UMAP where parameters were set, in my opinion, rather qualitatively because they were “found to be a good balance between separating dissimilar poses, while combining similar ones.” I worry that this description will not yield reproducible findings by other groups trying to use this method.

- We agree with the reviewer on the sentiment of qualitative assessment but we would like to point out that data of this type are so new and so unexplored that we are not yet in a space where we have sufficiently large and generalizable datasets in NHP to allow for more automated solutions. We chose to describe in in a way that was honest about the process. We note that this practice is common: many others perform the same tradeoff, although they seldom state it explicitly.

In fact, it is not clear what the UMAP achieves beyond the PCA, please clarify.

- An initial PCA is done primarily for computational efficiency’s sake. Essentially, most embedding solutions require pairwise comparison of data points making the computational complexity incredibly large. This problem can be reduced greatly with little loss of information by using PCA to impose sparsity on the data. A second benefit is that, since behavior is inherently lower dimensional than the data acquired, PCA additionally helps with noise reduction in the estimates.

- We have added the following explanation to the manuscript:

"Initial PCA dimensionality reduction was performed to reduce noise as well as to sparsen the data, which increases computational efficiency."

I was surprised to find that within-subject and between-subject module stability was analyzed via unpaired t-test when the data are collapsed and thus not independent.

- We note that our analysis explicitly makes the assumption that each session is an independent measure. That means that we are not well positioned to make across-subject claims, but of course the fact that we only have two subjects also means that. However, it is worth emphasizing that it does not risk false positives (Type I errors), just Type II errors. Nonetheless, this is a limitation of our approach, one that we should acknowledge more clearly.

"One major limitation of the present work is that we only recorded data in two subjects. As such we cannot make major claims about individual differences. Indeed, in our statistical analyses, we use the behavioral session, not the subject, as the unit of analysis. This approach risks reducing statistical power because it treats variance between subjects as no different from variance within. We believe that future work will mitigate some of these problems by including larger numbers of research subjects."

Similarly, a 2-way ANOVA on timescale across behavior does not seem appropriate. A multi-level generalized linear model with all between and within-subject factors included in the model would be more appropriate.

- See above. This is a limitation in this study, which we now acknowledge in the revised Discussion.

In general, I was disappointed with the lack of rodent behavioral tracking citations. I understand this is a macaque paper, but much has been learned from other species for behavioral elements/motifs and transitions to coordinated movement, or “motor sequences de novo” (Olveczky, Curr Op in Neurobio). Additionally, Luxem et al. could be cited https://elifesciences.org/articles/79305

- We have added these citations.

Minor
Line 24 “However, precise measure of behavior [in macaques] has generally been limited…”
Line 41 “macaque behaviors might not obey the same principles” as what?
Line 240 p=0.039

- These are all been fixed. (The comment about Line 41 no long applies as this text has been deleted).

Associate Editor: 1
Comments to the Author: There appears to be consensus from both Reviewers on the lack of clarity of (rationale for) the UMAP and perhaps some concerns about the choice of statistical tests for nested data.

- We now clarify the relative roles of the PCA and the UMAP.

- We now address the concerns about using nested tests.

Additionally, Reviewer 1 asking for more clarity on the rationale for alignment across datasets is justified.

- We now provide this.

Reviewer 1 also raises a fair point about tempering bold claims with some stated limitations of the present approach.

- We agree and have made these changes.

And finally Reviewer 2's suggestion to include rodent behavioral tracking citations seems reasonable.

- We now include this.

Overall, this is a valuable contribution to the study of naturalistic behavior in primates.

**Decision letter - OXFNSC-2022-003.R1**

| 02-Jun-2023  Dear Dr. Hayden,  It is a pleasure to accept your revised manuscript entitled "Hierarchical organization of rhesus macaque behavior" in its current form for publication in the Oxford Open Neuroscience. The comments of the reviewers who reviewed your manuscript are included at the foot of this letter.  Please note that this journal operates with transparent peer review. This means that  the full peer review history of your article will publish online alongside your article. This includes reviewer comments, editor decision letters, and your author responses.  Next steps You will receive an email from no-reply@scipris.com within roughly one week. This is your invitation to sign up for an account with SciPris, Oxford University Press’ author portal hosted by Aptara. You will need to create an account if you do not already hold one. Please register or log into your account and follow the online instructions which will guide you through signing your licence and paying the APC. The email and the portal have clearly signposted support options if you need any help during this process.  Please note that SciPris is a completely different system from ScholarOne, so your credentials to submit your manuscript here will not work there. Once you’ve created a SciPris account, you will be able to use it whenever you publish with Oxford Open Neuroscience or any OUP journal. Please note that OUP will only ever request payment for applicable fees be made via SciPris or to an OUP bank account. If you ever have concerns about the legitimacy of a request, please do not hesitate to contact a customer services agent via the SciPris portal or directly via oupsupport@scipris.com.  Thank you for your excellent contribution to the journal. On behalf of the Editors of the Oxford Open Neuroscience, we look forward to your continued contributions.  Sincerely, Dr. Alicia Izquierdo Senior Editor, Oxford Open Neuroscience aizquie@psych.ucla.edu  Reviewer: 1  Comments to the Author The authors have fully addressed my concerns. I appreciate the additional explanation on the use of UMAP and the alignment procedure. I can see now why these are valuable steps in the algorithm. I think this manuscript is a strong contribution to the growing field of computational ethology!  Reviewer: 2  Comments to the Author The authors have addressed all my concerns. Please note Figure 4 caption lettering is mislabeled.  Associate Editor Comments to the Author: Authors were responsive to reviewers and include appropriate edits in this revised manuscript. Figure 4 captions should be corrected in proof stage. | |
| --- | --- |
| **Date Sent:** | 02-Jun-2023 |
